# Meetings of Societies

**Published:** 1915-03

**Authors:** 


J. M. Fortescue-Brickdale.

### MEETINGS OF SOCIETIES.

### Bristol Medico-Chirurgical Society.

*January 13th*, 1915.

Dr. W. H. C. Newnham, President, in the Chair.

Dr. D. Alexander read a paper on **Epilepsy in Later Life.** *Abstract.*—Late onset is comparatively rare in true epilepsy, seizures of this character in the seventh decade being usually attributable to structural lesions of senescence and decay. Three cases are cited which are not to be accounted for by these antecedents. In one case there are petechiæ on the face and neck which suggest concomitant hemorrhages in the cortex.

Dr. J. Michell Clarke read a paper on **The Uses of Bleeding.** *Abstract.*—Chief uses of bleeding are: (1) to relieve an overdistended right heart; (2) to produce a local effect on some organ; (3) to act by the direct removal of a certain quantity of blood. (1) Mechanism of relief of right heart by venesection.

Importance of venous inflow into auricles for maintenance of systolic output of ventricles. Value of and indications for bleeding in mitral stenosis, in diseases of the lungs, in passive congestion of the liver, in apoplexy. (2) The use of bleeding in pericarditis, pleurisy, anuria of acute kidney diseases. (3) Venesection in uræmia, in chronically enlarged and failing hearts.

Mr. Hey Groves gave **An Account of some Surgical Experiences at the Base Hospitals in France,** illustrated by the epidiascope. *Abstract.*—Some of the voluntary hospitals in Paris. A day's work at a field hospital. Bringing the wounded into Paris after the battle of the Marne. The French military hospital at Val-de-Grace. Comments on certain groups of cases: head, spinal, chest, abdomen, bones and joints. Some of the Boulogne base hospitals. Problems of transport. Sepsis. Secondary hemorrhage. Gas gangrene.

J. Lacy Firth,
J. M. Fortescue-Brickdale,
*Acting Hon. Sec.*

---

